# Expression of the Rap1 Guanine Nucleotide Exchange Factor, MR-GEF, Is Altered in Individuals with Bipolar Disorder

**DOI:** 10.1371/journal.pone.0010392

**Published:** 2010-04-28

**Authors:** Angela Bithell, Tony Hsu, Apsara Kandanearatchi, Sabine Landau, Ian P. Everall, Ming T. Tsuang, Gursharan Chana, Brenda P. Williams

**Affiliations:** 1 Department of Psychological Medicine, Institute of Psychiatry, King's College London, London, United Kingdom; 2 Center for Behavioral Genomics, Department of Psychiatry, University of California San Diego, San Diego, California, United States of America; 3 Department of Biostatistics and Computing, Institute of Psychiatry, King's College London, London, United Kingdom; University of Muenster, Germany

## Abstract

In the rodent forebrain GABAergic neurons are generated from progenitor cells that express the transcription factors Dlx1 and Dlx2. The Rap-1 guanine nucleotide exchange factor, MR-GEF, is turned on by many of these developing GABAergic neurons. Expression of both Dlx1/2 and MR-GEF is retained in both adult mouse and human forebrain where, in human, decreased Dlx1 expression has been associated with psychosis. Using *in situ* hybridization studies we show that MR-GEF expression is significantly down-regulated in the forebrain of *Dlx1/2* double mutant mice suggesting that MR-GEF and Dlx1/2 form part of a common signalling pathway during GABAergic neuronal development. We therefore compared MR-GEF expression by *in situ* hybridization in individuals with major psychiatric disorders (schizophrenia, bipolar disorder, major depression) and control individuals. We observed a significant positive correlation between layers II and IV of the dorso-lateral prefrontal cortex (DLPFC) in the percentage of MR-GEF expressing neurons in individuals with bipolar disorder, but not in individuals with schizophrenia, major depressive disorder or in controls. Since MR-GEF encodes a Rap1 GEF able to activate G-protein signalling, we suggest that changes in MR-GEF expression could potentially influence neurotransmission.

## Introduction

Abnormalities in cortical GABAergic interneurons have been associated with psychiatric illness [1], [2]. In schizophrenia, both synthesis and reuptake of GABA are disrupted [3]–[7] and parvalbumin expressing chandelier neurons are preferentially affected [8]–[10]. Expression of GABA_A_ [11], [12], [13] and GABA_B_ [14], [15] receptors is also altered; possibly reflecting compensation by the brain for decreased GABA. Similar GABAergic neuronal changes occur in bipolar disorder [5], [15].

GABA_B_ receptors signal via guanine nucleotide binding proteins (G proteins) whose activity is regulated by guanine nucleotide exchange factors (GEFs) that activate signalling, and GTPase-activating proteins (GAPs) that inhibit signalling. We have shown that expression of a GEF, called mr-gef, is turned on in developing rodent GABAergic neurons [16]. The human homologue of this gene, called M-Ras-regulated GEF (*MR-GEF* or *RAPGEF5*), specifically activates the small GTPase Rap1 [17], known to regulate neurite outgrowth and synaptic transmission [18], [19]. Since many susceptibility genes for schizophrenia influence neurotransmission, synaptogenesis or synaptic plasticity [20], [21], it is possible that some of the observed GABAergic neuronal abnormalities in schizophrenia and bipolar disorder are associated with alterations in G protein signalling. Indeed, microarray analysis of gene expression differences associated with schizophrenia indicated that the most altered group of genes are those involved in presynaptic secretory function and in particular the regulator of G-protein signalling 4 gene (RGS4), encoding a GTPase activating protein, the expression of which was dramatically reduced in schizophrenia [22], [23].

Rodent forebrain GABAergic neurons are generated from progenitor cells that express the transcription factors Dlx1 and Dlx2 [24]. mr-gef is expressed by many of these developing GABAergic neurons and its expression is retained in adult rodent [16] and human brain [17]. Dlx1 and Dlx2 are also expressed widely throughout the adult mouse forebrain where they are involved in the migration, proliferation and neuronal sub-type specification of progenitor cells along the rostral migratory stream [25]–[27], and also in the maturation and survival of interneurons [28]. Little is known about Dlx expression in adult human brain; however, decreased Dlx1 expression in the thalamic mediodorsal nucleus, the principal source of thalamic afferents to the cortex that synapse on both pyramidal neurons and interneurons [29], has been associated with psychosis [30].

Here, we report that mr-gef expression is significantly down-regulated in mice that do not express Dlx1 and Dlx2, suggesting that they form part of a common signalling pathway during GABAergic neuronal development. Further, in the human DLPFC we observed a significant positive correlation between cortical layers II and IV in the percentage of MR-GEF expressing neurons in individuals with bipolar disorder, but not those with schizophrenia, major depressive disorder or controls. We further observed a significant positive correlation in the two-dimensional (2D) neuronal density between layers II and IV in individuals with bipolar disorder and schizophrenia, possibly reflecting a common pathology in these diseases.

## Methods

### Brain Tissue

Post-mortem brain sections (14 µm) from Brodmann area 9/46 (BA9/46) of the DLPFC were obtained from the Stanley Foundation Brain Consortium, an established collection of brain samples obtained with full consent of next of kin. The sample consisted of 60 subjects: 15 controls, 15 with schizophrenia, 15 with bipolar disorder, and 15 with major depressive disorder. None of these brains demonstrated evidence of neurodegenerative changes or other pathologic lesions. This study was performed blind to diagnosis on all 60 subjects, however 8 subjects were excluded from analysis due either to severe tissue damage (n = 1) or due to severe difficulty in locating landmarks to compare Nissl and MR-GEF slides because of poor tissue quality in the desired region (n = 7). A summary of demographic, clinical and histological information of the samples used in this study is given in Table S1. This study was carried out with the approval of the Institute of Psychiatry/South London and Maudsley NHS Trust Ethical Committee (ethical study number 153/01).

*Dlx1/2* mutant mouse brains were the kind gift of John Rubenstein and Stewart Anderson [24].

### Non-radioactive *in situ* hybridisation and Nissl staining

Sense and anti-sense probes were designed to specifically target a region from within the open reading frame (ORF) of the rodent (mr-gef) [16] or human (MR-GEF) transcript. The human MR-GEF mRNA comprises a short 5′ untranslated region (UTR), an ORF of 1740 bp and a long 3′ UTR of more than 3 kb that generates a single transcript (see Figure S1) [17].

Additional cDNAs used for riboprobe synthesis were Lhx6 (Lhx6 cDNA was a gift from Vassilis Pachnis and Maria Grigoriou, NIMR, London, UK) and Dlx1 (Dlx1 cDNA was a gift from John Rubenstein, University College of San Francisco).

Sections were fixed for 10 minutes in cold 4% paraformaldehyde in 0.1 M phosphate buffer before being washed in Depc-PBS (diethylpyrocarbonate treated phosphate buffered saline), incubated in acetylation solution for 10 minutes and permeabilised by incubation in 1% Triton X-100 (Sigma) for 5 minutes (human tissue) or 30 minutes (mouse tissue). After further washes in Depc-PBS, slides were placed in a hybridisation chamber humidified with 50% formamide and 5xSSC. Sections were pre-hybridised in 500 µl hybridisation buffer (50% formamide, 5xDepc-SSC, 5x Denhardts, 0.25 mg/ml yeast RNA, 0.5 mg/ml herring sperm DNA) for 2–6 hours at room temperature. DIG-labelled RNA probes (100–200 ng/ml diluted in hybridisation buffer) were denatured at 80°C for 5 minutes then cooled on ice. The probe/hybridisation buffer mix was pipetted onto sections and the slides covered with glass coverslips. Hybridisation was carried out at 65°C overnight in a sealed, humidified hybridisation chamber. Sections then went through a series of washes (0.2xSSC at 65°C for 1 hour; 0.2xSSC for 5 mins at room temperature; 0.1 M Tris pH 7.5/150 mM NaCl for 5 mins at room temperature) before being blocked in 10% heat inactivated sheep serum (Sigma, diluted in 0.1 M Tris pH 7.5/150 mM NaCl) for 1–3 hours at room temperature. To detect the DIG-labelled riboprobes, 500 µl of anti-DIG Fab-AP antibody (Roche, diluted 1∶5000) was added to each slide and incubated overnight at 4°C in a humidified chamber. Slides were then washed 3×5 minutes in 0.1 M Tris pH 7.5/150 mM NaCl, followed by 5 minutes in 0.1 M Tris pH 9.5, 100 mM NaCl, 50 mM MgCl2 with 0.24 mg/ml levamisole (Sigma) to inhibit endogenous phosphatase activity. Bound probes were visualised by incubation in the dark in colour solution (50 ng/ml NBT [Roche] and 75 ng/ml BCIP [Merck] with levamisole). Reactions were stopped in water or 1x TE (sense and anti-sense reactions were stopped at the same time). In all cases sense probes showed no specific labelling (see Figure S1). Slides were mounted in Faramount aqueous mounting medium (Dako) and then either photographed using a Nikon Eclipse microscope and processed with Lucia G and Adobe Photoshop imaging software, for mouse tissue, or analysed as described below for human post-mortem tissue.

For human post-mortem analyses, Nissl staining on adjacent sections to those processed for non-radioactive *in situ* hybridisation was performed as described previously [31].

### Image analysis and counting

Tissue sections were viewed using a 20x objective on a Leica BMLB microscope equipped with a Hitatchi colour camera and a Marzhauser x-, y-motorised stage. Similar regions in area BA46 were selected on each slide based on cytoarchitecture. Obvious landmarks were chosen (blood vessels, contour of tissue) in Nissl stained sections to allow the same region to be detected on adjacent MR-GEF stained sections. Composite images from the pial surface to the grey/white matter border were generated as previously described [31], [32]. The size of each composite image was standard in the x-axis (600 µm) but varied in the y-axis according to the cortical thickness. Layers were identified, manually inserted onto the Nissl image and then overlaid onto MR-GEF images to allow layer identification. The soma of stained cells was outlined manually to ensure all cell outlines were accurate and all non-neuronal material such as blood vessels were excluded. Two sections were analysed for each case and from each composite image two fields were chosen for counting. Within each field the total number of neurons within our two-dimensional plane were counted using the Nissl stained sections. MR-GEF labelled/Nissl stained cells were counted from an identical region on the adjacent section and their percentage of the total Nissl stained population calculated. Somal sizes were measured by a computer algorithm within Image Pro Plus 4.0, converting pixels covered by cell outlines to square microns by using a calibration factor for the x20 objective used.

### Statistical analysis

#### Identification of Adjustment Variables

Post-mortem and demographic variables were included as co-variates where they were found to correlate with outcome at p<0.05. This included assessment of medications via lifetime fluphenazine equivalents, substance abuse and use of ethanol as supplied to us by the Stanley Foundation. In addition, in order to control for potential differential shrinkage of layers 2 and 4 for individual cases we calculated the percentage of the cortical width that each case represented and analysed differences between groups at p<0.05. We found no significant differences in the proportions of layer II and IV to the whole cortical width between any of the psychiatric groups versus controls.

#### Group Comparisons

Statistical analysis was carried out using a one-way analysis of variance (ANOVA) to test for differences in estimates between psychiatric and control groups using SPSS 18.0 (SPSS, Inc.) at a bonferroni adjusted significance level of p<0.05/2 = 0.025. We determined that this analysis was appropriate after showing that our data was normally distributed for neuronal somal size; both mean and median neuronal somal size was analysed and no significant difference were observed between groups. The percentage of neurons that expressed MR-GEF and the 2D areal density of neurons in layers II and IV was analysed in sections from patient versus control groups. We also carried out a pearson correlation analysis between layers II and IV for the percentage of MR-GEF expressing neurons as well as total neurons (expressing and non-expressing) within groups. The main objective of the statistical analysis was to compare the outcome in each of the patient groups compared with the control group and to identify any relationships that may be specific to the major psychiatric disorder under investigation.

## Results

### mr-gef expression is perturbed in the *Dlx1/2* mutant forebrain

**Figure 1:**
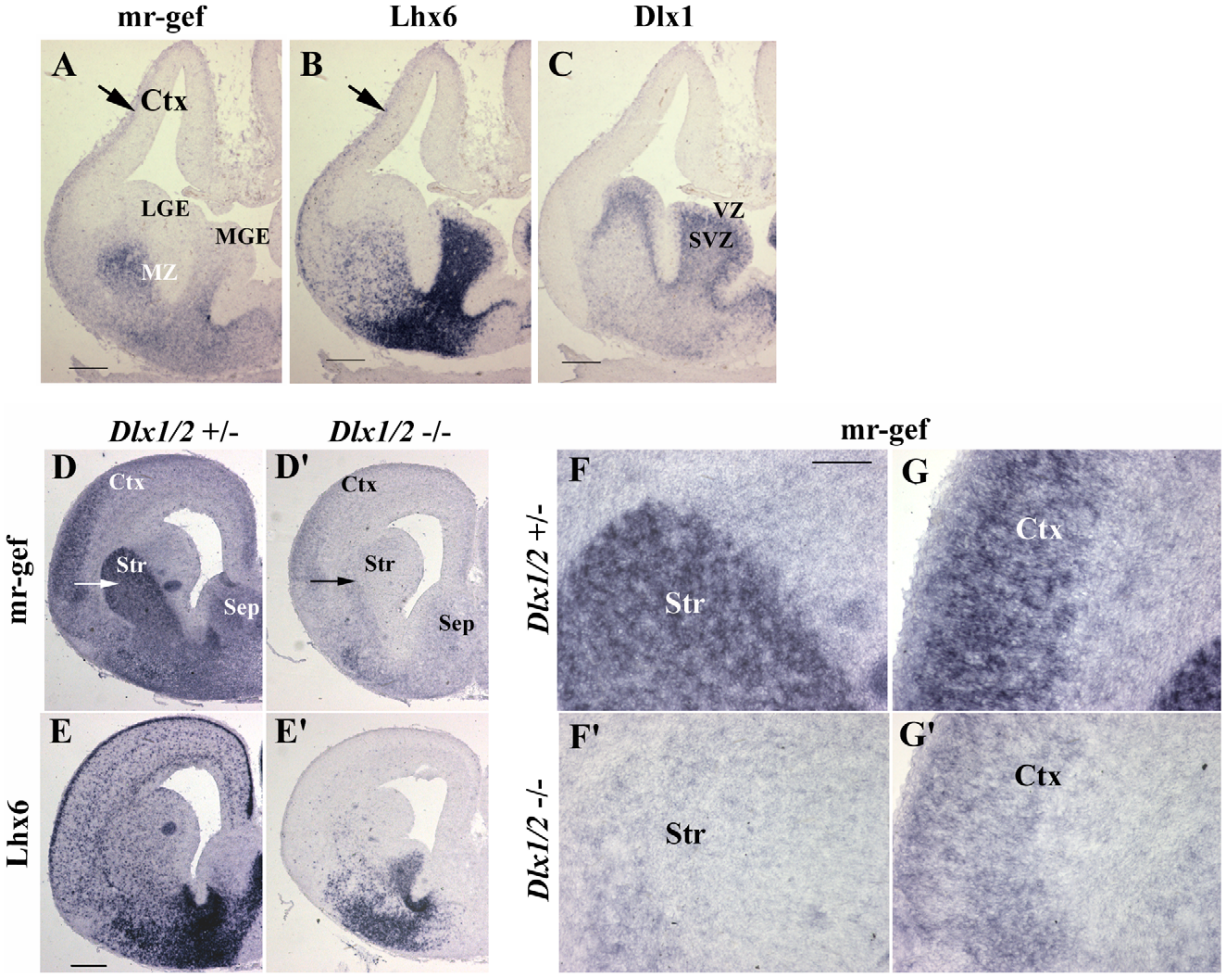
Association between mr-gef and Dlx1 expression. Regional expression of mr-gef, Lhx6 and Dlx1 in the E14.5 mouse ventral telencephalon (A–C). mr-gef and Lhx6 are expressed in the SVZ and mantle of the MGE (A,B respectively). mr-gef is expressed in regions adjacent to those in the VZ and SVZ expressing Dlx1 (A and C respectively) and in a band of cells reaching up into the Ctx (arrow A). A similar mr-gef expression pattern is observed in the ventral telencephalon in the *Dlx1/2* heterozygote (+/−, D), with highest levels in the striatum (white arrow) and septum. Higher power magnification of striatum and cortex is shown in F and G respectively. In the *Dlx1/2* double mutant telencephalon, mr-gef expression is greatly reduced, particularly in the striatum (black arrow, D'). Expression in the mutant striatum and cortex is shown at higher power in F' and G' respectively. For comparison, Lhx6 expression is shown in adjacent sections from the same heterozygote and mutant telencephalon (E and E' respectively), with a loss of Lhx6 expression in the cortex of the mutant (E'). Abbreviations: VZ, ventricular zone; SVZ, subventricular zone; MGE, medial ganglionic eminence; Str, striatum; Ctx, cortex; Sep, septum. Scale bars: 250 µm.

During development, mr-gef expression showed a striking overlap with the LIM-homeobox gene, Lhx6, but a reciprocal pattern of expression with the transcription factor Dlx1 (Figure 1A–C). Lhx6 is expressed by, and required for, the migration of GABAergic neurons during development [33], while Dlx1, together with its family member Dlx2, is necessary for the correct development of many GABAergic neurons [24]. Both Lhx6 and mr-gef were expressed in the subventricular zone (SVZ) of the striatal anlage (termed the lateral and medial ganglionic eminences, LGE and MGE, respectively, in rodent); in the mantle zone (MZ) composed of young neurons; and in a band of cells reaching up into the cortex (Figure 1 arrows in A, B ) composed of migrating GABAergic neurons [33]. In contrast, Dlx1 expression was restricted to the ventricular zone (VZ) and the SVZ. Thus, mr-gef was expressed in cells lying directly adjacent to Dlx1 expressing cells (Figure 1, compare A with C), suggesting that mr-gef expression was turned on in young neurons as they differentiated from Dlx1 (and Dlx2) expressing progenitor cells.

To determine whether Dlx1 and 2 were required for mr-gef expression, we compared mr-gef expression in the forebrain of mice where both *Dlx1* and *Dlx2* have been knocked out (*Dlx1/2 −/−* mice) with expression in heterozygote littermates as controls (Figure 1 D, D' – G, G'). In the E16.5 *Dlx1/2* heterozygote forebrain intense mr-gef expression was observed in the striatum (str), cortical plate (ctx), septum (sep) and pallidum (Figure 1D and at higher magnification in F and G). In sharp contrast, mr-gef expression in the *Dlx1/2 −/−* forebrain was dramatically reduced within the striatum (Figure 1, compare D, D′ and F, F′) and cortical plate (Figure 1, compare D, D′ and G, G′). This latter observation could be explained by a failure of some ventrally-derived mr-gef expressing cells to migrate into the cortex, since GABAergic neuronal development is disrupted in these mice and migration of Lhx6-expressing GABAergic neurons into the cortex is inhibited [24], [25]. This migration failure can be observed by comparing Lhx6 expression in the heterozygote and mutant forebrain (Figure 1 E and E′ respectively). We assume that the remaining mr-gef expression in the cortex is regulated by other transcription factors [16]. Thus, these studies suggest that mr-gef expression in the majority of striatal neurons, and in a subpopulation of cortical plate neurons, requires *Dlx1* and/or *Dlx2*.

### MR-GEF & NISSL expression in human DLPFC layer II and layer IV

Our observed reduction in striatal and cortical mr-gef expression in *Dlx1/2 −/−* mice, together with the previously reported observation that Dlx1 expression is decreased in the mediodorsal thalamic nucleus (MDTN) in patients with psychosis [30], prompted us to determine whether MR-GEF expression was perturbed in individuals with psychiatric disorders. The MDTN is the major source of excitatory thalamic input to the DLPFC and a decrease in the neuronal number and volume of this nucleus has been reported in schizophrenia [34], [35]. Although the pyramidal neurons in cortical layers III and V are the primary targets of these thalamic afferents, these pyramidal neurons also require GABAergic input in order to function properly. Indeed, GABAergic neurons may also receive a direct input from thalamic afferents [29]. This intimate relationship between GABAergic interneurons, pyramidal neurons and neuronal input from the MDTN, suggests that the functional impairment of any one of these cell types could cause dysregulation of excitatory input and output from this brain region.

**Figure 2:**
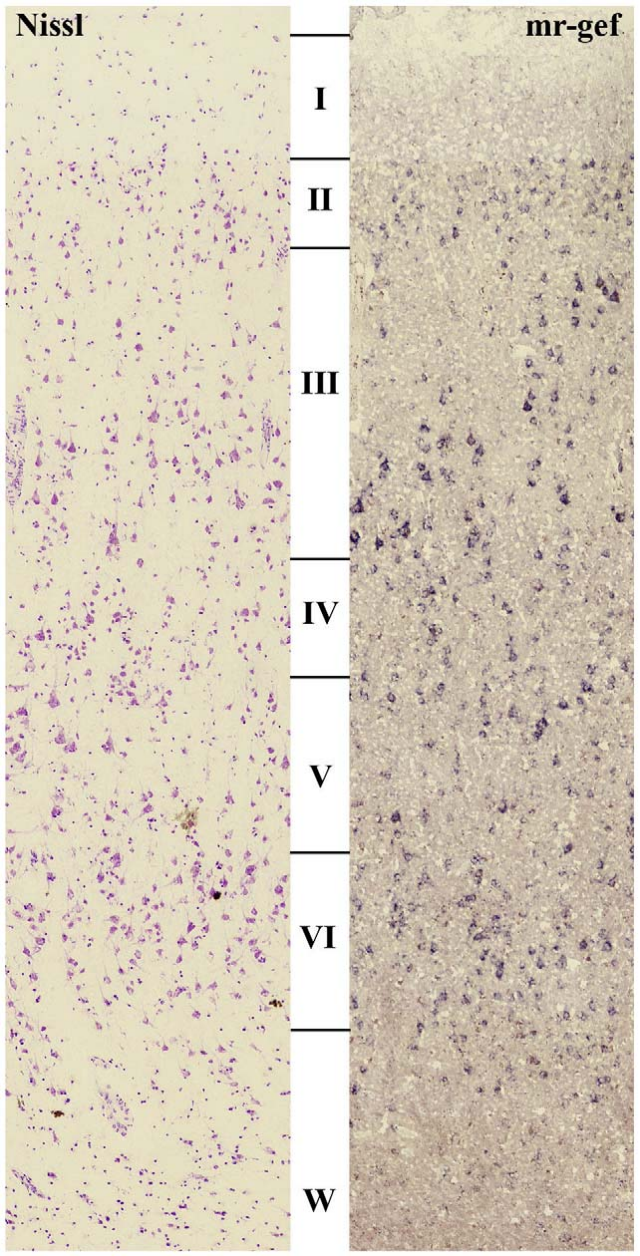
MR-GEF labelled and Nissl stained sections. Comparison of Nissl stained and MR-GEF labelled serial sections showing that MR-GEF is expressed by cells throughout the cortex and is not confined to GABAergic neurons.

**Figure 3:**
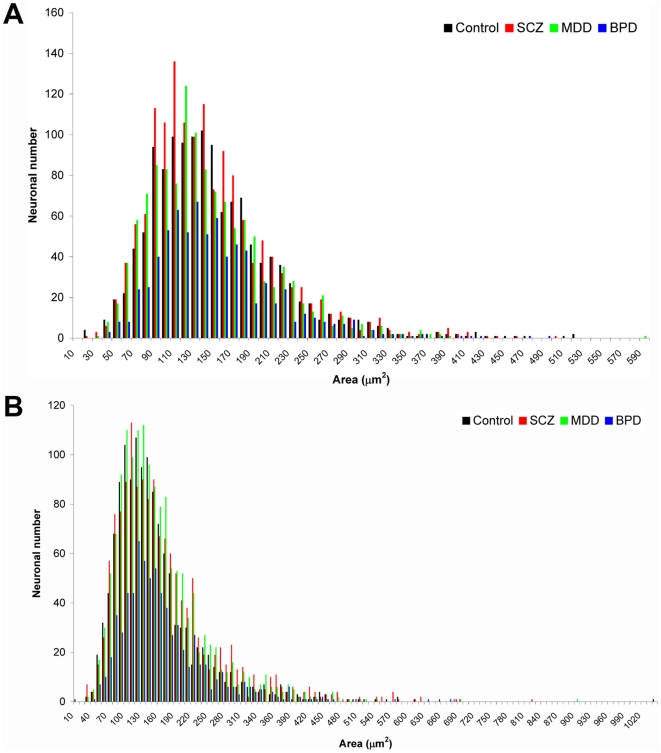
Distribution of MR-GEF-expressing neuronal sizes by group for cortical layers II and IV. Comparison of the range of somal sizes of MR-GEF expressing neurons in layer II (A) and layer IV (B) of the DLPFC. Abbreviations: BPD, bipolar disorder; MDD, major depressive disorder; SCZ, schizophrenia.

When comparing Nissl stained and MR-GEF labelled serial sections it was apparent that MR-GEF expression in the human DLPFC was not restricted to GABAergic neurons (Figure 2). However, the developmental relationship observed between Dlx1, mr-gef and GABAergic neurons prompted us to focus our initial MR-GEF expression study on layers II and IV of the DLPFC since these layers are rich in a variety of GABAergic neuronal subtypes [10], [36], [37]. Because these layers also include small pyramidal neurons, it is possible that the types of neurons expressing MR-GEF could vary between patient groups and/or between patient and control groups. We therefore compared the somal sizes of neurons expressing MR-GEF in layers II and IV from each group (Figure 3). We reasoned that a shift toward larger somal sizes would indicate that more pyramidal neurons were expressing MR-GEF while a shift toward smaller somal sizes would indicate that more GABAergic neurons expressed this gene. The range of sizes observed was very similar across all groups (see Figure 3). However, in both layer II and IV there appeared to be fewer neurons of all sizes in individuals with bipolar disease, and in layer II there appeared to be more neurons of all sizes in individuals with schizophrenia (Figure 3). This difference was not reflected in our statistical analysis looking at both mean and median somal sizes, where no significant difference was observed between groups.

When the percentage of neurons expressing MR-GEF was calculated and compared across all groups and layers no significant difference was observed. Neither was there any significant difference observed in the 2D density of neurons between patient and control groups.

### Correlations in MR-GEF expression and neuronal density between layers II and IV

Due to the intimate association between interneurons and pyramidal neurons within the different cortical layers, we assessed the correlation between the percentage of MR-GEF expressing neurons in layers II and IV across the control and patient groups.

We observed a significant positive correlation in the percentage of neurons expressing MR-GEF between layers II and IV in individuals with bipolar disorder (r = 0.704 and p = 0.034) No correlation was observed in individuals with schizophrenia, major depressive disorder or in controls. We also applied the same analysis to the 2D density counts between layers II and IV and observed a significant positive correlation in individuals with schizophrenia (r = 0.586, p = 0.028) and bipolar disorder (r = 0.732. p = 0.025) while no correlation was observed in major depressive disorder or in controls.

## Discussion

During rodent forebrain development many GABAergic neurons express the Rap1 GEF, mr-gef [16]. Here we showed that mr-gef expression is dramatically reduced in the striatum and cortical plate of *Dlx1/2 −/−* mice. These observations suggest mr-gef and Dlx1/2 form part of a common pathway during GABAergic neuronal development. Both mr-gef and Dlx1 expression is retained in adult mouse [16], [26] and human [17], [30] brain, where Dlx1 has been shown to be decreased in individuals with psychosis [15]. Here we report that the percentage of neurons expressing the human homologue of *mr-gef* shows a significant positive correlation between layers II and IV in individuals with bipolar disorder but not in those with schizophrenia, major depressive disorder or in controls. This observation could be explained by perturbation of cortical organisation and/or communication associated with bipolar disorder. In addition, individuals with bipolar disorder and those with schizophrenia show a significant positive correlation in 2D neuronal density between Layers II and IV, supporting the growing body of evidence reporting a shared pathology between these diseases [38], [39].

In humans, neocortical GABAergic interneurons are derived from Dlx-expressing populations in both the neocortex and the ganglionic eminences (GE) [40], whilst those of the thalamus are derived from the GE; a migratory pathway not found in rodents [41]. In rodents most, if not all, of the GABAergic neurons of the cortex migrate in from the GE [24]. We have observed that mr-gef expression is dramatically reduced in the striatum and cortex of *Dlx1/2 −/−* mice. This could be explained by a failure of mr-gef-expressing cells to develop and migrate appropriately during development, since Dlx1 and -2 are known to direct the correct development and migration of many GABAergic neurons within the developing forebrain [24]. Dlx expression and function in the adult human cerebral cortex has not been investigated, however, in rodent, expression is observed throughout the cerebral cortex, with expression being highest in layers II and III [26] where mr-gef is also highly expressed (AB, BPW unpublished observations) suggesting that these proteins also have the potential to be part of a common signalling pathway in the adult brain.

Within the cerebral cortex, different GABAergic neuronal subtypes innervate particular domains on pyramidal neurons and have a specific function in regulating pyramidal neuron activity [42]. Recent reports suggest that only a sub-population of GABAergic neurons is affected in schizophrenia - the PV-expressing neurons [8], [9]. These neurons do not appear to be reduced in number but show decreased expression of critical genes, including GAD67 and GAT1, suggesting that they are functionally impaired. Since GABAergic neurons form a network of connections throughout the cortex and all influence pyramidal cell function, it is highly conceivable that functional impairment of a particular subpopulation will have a knock-on effect on the population as a whole. Although speculative, if Rap1 signalling is involved in synaptic transmission, then an increase in MR-GEF expressing neurons (a positive regulator of G protein signalling) may represent an attempt by cortical neurons to overcome their inherent signalling deficit, as suggested for the observed increase in GABA receptor expression [9]. In humans, decreased Dlx1 expression in the mediodorsal thalamic nucleus, the principal source of thalamocortical connections that selectively terminate in layers III and IV of the PFC, is associated with psychosis [30]. Indeed, abnormalities in incoming thalamic afferents that synapse onto pyramidal neurons within layer III and V could result in dysregulation of excitatory input and output from the DLPFC. In addition, to function properly, these pyramidal neurons also require inhibitory input from GABAergic neurons, some of which will reside within layers II and IV. Thus, dysregulation of any part of this neuronal network could contribute to functional impairment of the region.

When we assessed the correlation in the percentage of MR-GEF expressing neurons between layers II and IV across patient and control groups, we found a significant positive relationship for the percentage of MR-GEF expressing neurons between the two layers in bipolar disorder, possibly suggesting that communication between these two layers is altered in this disease. In addition, the observed significant positive correlation between layers II and IV in 2D neuronal density in individuals with bipolar disorder and schizophrenia may reflect a common pathology in these diseases.

Our observed correlations in both the percentage of MR-GEF expressing neurons and in 2D neuronal density between layers II and IV in bipolar disorder support a growing body of evidence for defects in cortical organisation and communication in this disease. Moreover, since MR-GEF encodes a Rap1 GEF able to activate G-protein signalling, we suggest that changes in MR-GEF expression could potentially influence neurotransmission.

## Supporting Information

Table S1 Demographic, clinical and histological data for the 52 analysed patient cases. * One case has no pH information, number given is average of remaining 11 cases. Abbreviations: SCZ, schizophrenia; BPD, Bipolar Disorder; MDD, major depressive disorder; PMI, post-mortem interval.
(0.03 MB DOC)

Figure S1 MR-GEF in situ hybridation on human sections. Cartoon of the human MR-GEF mRNA comprising a short 5′ untranslated region (UTR), an open reading frame (ORF) of 1740 bp and a long 3′ UTR of more than 3 kb (A). Two different probes specific for MR-GEF were designed either to target a portion of the ORF of approximately 800 bp or to target a portion of the 3′ UTR of approximately 1.2 kb (A). In both cases sense probes gave no specific labelling whilst anti-sense gave a specific signal with very little background labelling. A representative image of labelling with sense and anti-sense MR-GEF ORF probes on adjacent sections is shown (B). All experiments described in the text were carried out using MR-GEF ORF sense and anti-sense probes. Scale bar  = 200 µm.
(2.98 MB TIF)

## References

[1] Costa E, Davis JM, Dong E, Grayson DR, Guidotti A (2004). A GABAergic cortical deficit dominates schizophrenia pathophysiology.. Crit Rev Neurobiol.

[2] Lewis DA, Hashimoto T, Volk DW (2005). Cortical inhibitory neurons and schizophrenia.. Nat Reviews.

[3] Akbarian S, Kim JJ, Potkin SG, Hagman JO, Tafazzoli A (1995). Gene expression for glutamic acid decarboxylase is reduced without loss of neurons in prefrontal cortex of schizophrenics.. Arch Gen Psychiatry.

[4] Woo TU, Whitehead RE, Melchitzky DS, Lewis DA (1998). A subclass of prefrontal gamma-aminobutyric acid axon terminals are selectively altered in schizophrenia.. Proc Natl Acad Sci U S A.

[5] Guidotti A, Auta J, Davis JM, Di-Giorgi-Gerevini V, Dwivedi Y (2000). Decrease in reelin and glutamic acid decarboxylase67 (GAD67) expression in schizophrenia and bipolar disorder: a post-mortem brain study.. Arch Gen Psychiatry.

[6] Volk DW, Austin MC, Pierri JN, Sampson AR, Lewis DA (2000). Decreased glutamic acid decarboxylase67 messenger RNA expression in a subset of prefrontal cortical gamma-aminobutyric acid neurons in subjects with schizophrenia.. Arch Gen Psychiatry.

[7] Volk D, Austin M, Pierri J, Sampson A, Lewis D (2001). GABA transporter-1 mRNA in the prefrontal cortex in schizophrenia: decreased expression in a subset of neurons.. Am J Psychiatry.

[8] Beasley CL, Reynolds GP (1997). Parvalbumin-immunoreactive neurons are reduced in the prefrontal cortex of schizophrenics.. Schizophr Res.

[9] Lewis DA (2000). GABAergic local circuit neurons and prefrontal cortical dysfunction in schizophrenia.. Brain Res Brain Res Rev.

[10] Hashimoto T, Volk DW, Eggan SM, Mirnics K, Pierri JN (2003). Gene expression deficits in a subclass of GABA neurons in the prefrontal cortex of subjects with schizophrenia.. J Neurosci.

[11] Benes FM, Vincent SL, Marie A, Khan Y (1996). Up-regulation of GABAA receptor binding on neurons of the prefrontal cortex in schizophrenic subjects.. Neuroscience.

[12] Dean B, Hussain T, Hayes W, Scarr E, Kitsoulis S (1999). Changes in serotonin2A and GABA(A) receptors in schizophrenia: studies on the human dorsolateral prefrontal cortex.. J Neurochem.

[13] Lo WS, Lau CF, Xuan Z, Chan CF, Feng GY (2004). Association of SNPs and haplotypes in GABAA receptor beta2 gene with schizophrenia.. Mol Psychiatry.

[14] Mizukami K, Ishikawa M, Hidaka S, Iwakiri M, Sasaki M (2002). Immunohistochemical localization of GABA_B_ receptor in the entorhinal cortex and inferior temporal cortex of schizophrenic brain.. Prog Neuropsychopharmacol Biol Psychiatry.

[15] Ishikawa M, Mizukami K, Iwakiri M, Asada T (2005). Immunohistochemical and immunoblot analysis of gamma-aminobutyric acid B receptor in the prefrontal cortex of subjects with schizophrenia and bipolar disorder.. Neurosci Lett.

[16] Bithell A, Alberta J, Hornby F, Stiles SD, Williams BP (2003). Expression of the guanine nucleotide exchange factor, mr-gef, is regulated during the differentiation of specific subsets of telencephalic neurons.. Brain Res Dev Brain Res.

[17] Rebhun JF, Castro AF, Quilliam LA (2000). Identification of guanine nucleotide exchange factors (GEFs) for the Rap1 GTPase. Regulation of MR-GEF by M-Ras-GTP interaction.. J Biol Chem.

[18] Morozov A, Muzzio IA, Bourtchouladze R, Van-Strien N, Lapidus K (2003). Rap1 couples cAMP signaling to a distinct pool of p42/44MAPK regulating excitability, synaptic plasticity, learning, and memory.. Neuron.

[19] Xie Z, Huganir RL, Penzes P (2005). Activity-dependent dendritic spine structural plasticity is regulated by small GTPase Rap1 and its target AF-6.. Neuron.

[20] Harrison PJ, Weinberger DR (2005). Schizophrenia genes, gene expression, and neuropathology: on the matter of their convergence.. Mol Psychiatry.

[21] Straub RE, Lipska BK, Egan MF, Goldberg TE, Callicott JH (2007). Allelic variation in GAD1 (GAD67) is associated with schizophrenia and influences cortical function and gene expression.. Mol Psychiatry.

[22] Mirnics K, Middleton FA, Lewis DA, Levitt P (2001). Analysis of complex brain disorders with gene expression microarrays: schizophrenia as a disease of the synapse.. TINS.

[23] Talkowski ME, Chowdari K, Lewis DA, Nimgaonkar VL (2006). Can RGS4 Polymorphisms Be Viewed as Credible Risk Factors for Schizophrenia? A Critical Review of the Evidence.. Schizophr Bull.

[24] Anderson S, Mione M, Yun K, Rubenstein JL (1999). Differential origins of neocortical projection and local circuit neurons: role of Dlx genes in neocortical interneuronogenesis.. Cereb Cortex.

[25] Stuhmer T, Puelles L, Ekker M, Rubenstein JL (2002). Expression from a Dlx gene enhancer marks adult mouse cortical GABAergic neurons. Cereb.. Cortex.

[26] Saino-Saito S, Berlin R, Baker H (2003). Dlx-1 and Dlx-2 expression in the adult mouse brain: relationship to dopaminergic phenotypic regulation.. J Comp Neurol.

[27] Brill MS, Snapyan M, Wohlfrom H, Ninkovic J, Jawerka M (2008). A dlx2- and pax6-dependent transcriptional code for periglomerular neuron specification in the adult olfactory bulb.. J Neurosci.

[28] Cobos I, Calcagnotto ME, Vilaythong AJ, Thwin MT, Noebels JL (2005). Mice lacking Dlx1 show subtype-specific loss of interneurons, reduced inhibition and epilepsy.. Nat Neurosci.

[29] Rotaru DC, Barrionuevo G, Sesack SR (2005). Mediodorsal thalamic afferents to layer III of the rat prefrontal cortex: synaptic relationships to subclasses of interneurons.. J Comp Neurol.

[30] Kromkamp M, Uylings HB, Smidt MP, Hellemons AJ, Burbach JP (2003). Decreased thalamic expression of the homeobox gene DLX1 in psychosis.. Arch Gen Psychiatry.

[31] Chana G, Landau S, Beasley C, Everall IP, Cotter D (2003). Two-dimensional assessment of cytoarchitecture in the anterior cingulate cortex in major depressive disorder, bipolar disorder, and schizophrenia: evidence for decreased neuronal somal size and increased neuronal density.. Biol Psychiatry.

[32] Cotter D, Mackay D, Chana G, Beasley C, Landau S (2002). Reduced neuronal size and glial cell density in area 9 of the dorsolateral prefrontal cortex in subjects with major depressive disorder.. Cereb Cortex.

[33] Lavdas AA, Grigoriou M, Pachnis V, Parnavelas JG (1999). The medial ganglionic eminence gives rise to a population of early neurons in the developing cerebral cortex.. J Neurosci.

[34] Popken GJ, Bunney WE, Potkin SG, Jones EG (2000). Subnucleus-specific loss of neurons in medial thalamus of schizophrenics.. Proc Natl Acad Sci USA.

[35] Byne W, Buchsbaum MS, Mattiace LA, Hazlett eA, Kemether E, Elhakem SL (2002). Postmortem assessment of thalamic nuclear volumes in subjects with schizophrenia.. Am J Psychiatry.

[36] Beasley CL, Zhang ZJ, Patten I, Reynolds GP (2002). Selective deficits in prefrontal cortical GABAergic neurons in schizophrenia defined by the presence of calcium-binding proteins.. Biol Psychiatry.

[37] Chance SA, Walker M, Crow TJ (2005). Reduced density of calbindin-immunoreactive interneurons in the planum temporale in schizophrenia.. Brain Res.

[38] Murray RM, Sham P, Van Os J, Zanelli J, Cannon M (2004). A developmental model for similarities and dissimilarities between schizophrenia and bipolar disorder.. Schizophr Res.

[39] Craddock N, O'Donovan MC, Owen MJ (2006). Genes for schizophrenia and bipolar disorder? Implications for psychiatric nosology.. Schizophr Bull.

[40] Letinic K, Zoncu R, Rakic P (2002). Origin of GABAergic neurons in the human neocortex.. Nature.

[41] Letinic K, Rakic P (2001). Telencephalic origin of human thalamic GABAergic neurons.. Nature Neurosci.

[42] Somogyi P, Tamás G, Lujan R, Buhl EH (1998). Salient features of synaptic organisation in the cerebral cortex.. Brain Res Brain Res Rev.

