# Supplementary material for: Expression of the Rap1 Guanine Nucleotide Exchange Factor, MR-GEF, Is Altered in Individuals with Bipolar Disorder
Source: PLoS One. 2010 Apr 28;5(4):e10392. doi: 10.1371/journal.pone.0010392 (PMC2861006; doi:10.1371/journal.pone.0010392)
Supplement: Table S1 — Demographic, clinical and histological data for the 52 analysed patient cases. * One case has no pH information, number given is average of remaining 11 cases. Abbreviations: SCZ, schizophrenia; BPD, Bipolar Disorder; MDD, major depressive disorder; PMI, post-mortem interval. (0.03 MB DOC) [file pone.0010392.s001.doc]

| Tissue | Cases | Age (years) | Gender (Male:Female) | pH | PMI (hours) | Medication (Yes : No) | Psychosis (Yes : No) | Cause of death (suicide : non suicide) |
| --- | --- | --- | --- | --- | --- | --- | --- | --- |
| Control | 14 | 48.4 | 8M:6F | 6.3 | 24.7 | 0Y:14N | 0Y:14N | 0S:14NS |
| SCZ | 14 | 43.6 | 9M:5F | 6.2 | 34.2 | 13Y:1N | 14Y:0N | 4S:10NS |
| BPD | 12 | 42.4 | 7M:5F | 6.2 | 34.0 | 10Y:2N | 9Y:3N | 7S:5NS |
| MDD | 12 | 46.1 | 7M:5F | 6.2* | 26.3 | 0Y:12N | 0Y:12N | 6S:6NS |
