# Supplementary figures and images for: Expression of the Rap1 Guanine Nucleotide Exchange Factor, MR-GEF, Is Altered in Individuals with Bipolar Disorder
Source: PLoS One. 2010 Apr 28;5(4):e10392. doi: 10.1371/journal.pone.0010392 (PMC2861006; doi:10.1371/journal.pone.0010392)

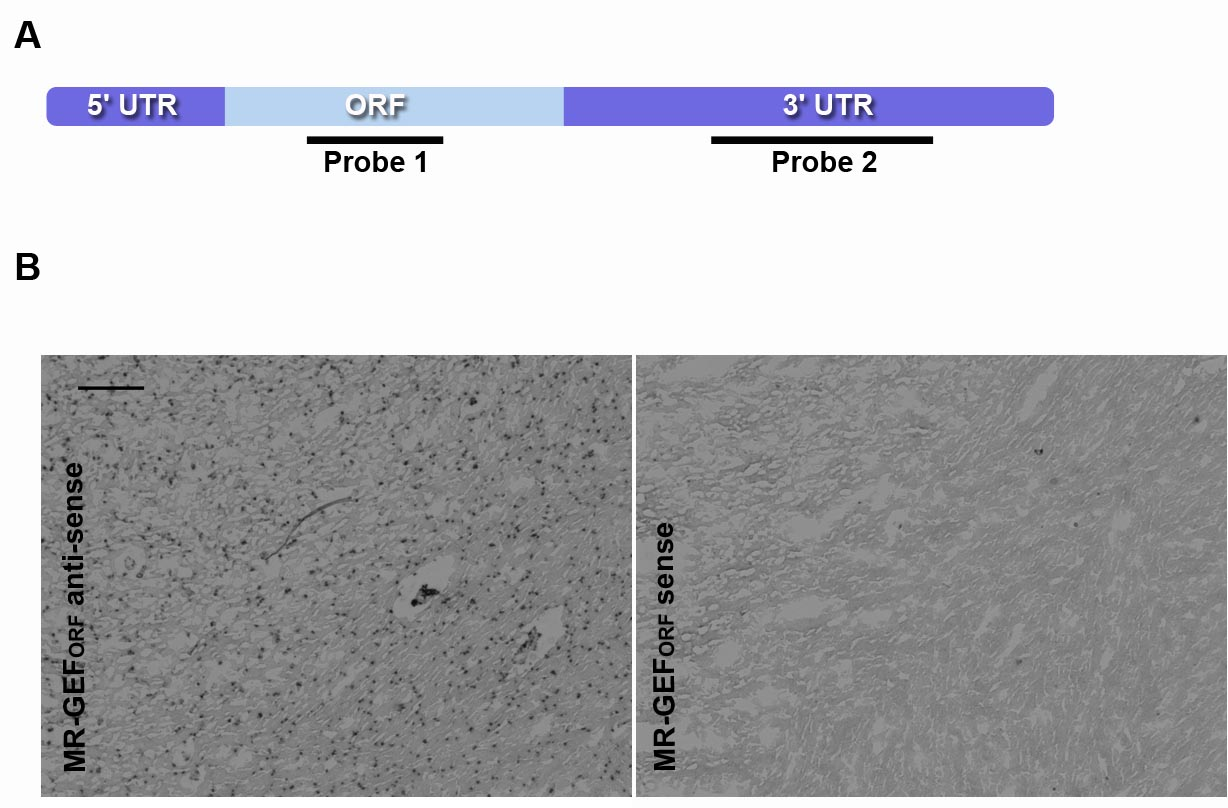

Supplement: Figure S1 — MR-GEF in situ hybridation on human sections. Cartoon of the human MR-GEF mRNA comprising a short 5′ untranslated region (UTR), an open reading frame (ORF) of 1740 bp and a long 3′ UTR of more than 3 kb (A). Two different probes specific for MR-GEF were designed either to target a portion of the ORF of approximately 800 bp or to target a portion of the 3′ UTR of approximately 1.2 kb (A). In both cases sense probes gave no specific labelling whilst anti-sense gave a specific signal with very little background labelling. A representative image of labelling with sense and anti-sense MR-GEF ORF probes on adjacent sections is shown (B). All experiments described in the text were carried out using MR-GEF ORF sense and anti-sense probes. Scale bar = 200 µm. (2.98 MB TIF) [file pone.0010392.s002.tif]
